# Internet Impact on the Insertion of Genitourinary Tract Foreign Bodies in Childhood

**DOI:** 10.1155/2012/102156

**Published:** 2012-09-11

**Authors:** Xenophon Sinopidis, Vasileios Alexopoulos, Antonios Panagidis, Alexandra Ziova, Anastasia Varvarigou, George Georgiou

**Affiliations:** ^1^Department of Pediatric Surgery, Karamandanion Children's Hospital, 26331 Patras, Greece; ^2^Department of Pediatrics, Patras University Hospital, 26504 Patras, Greece

## Abstract

Foreign body self-insertion into the urethra is an uncommon paraphilia. Variety in object form, motivation, clinical presentation, complications, and treatment options is a rule. In childhood it is very rare, and it is attributed to curiosity or mental disorders so far. However, the internet impact on daily life of all age groups has created a new category of sexual behavior in childhood and adolescence, the “internet induced paraphilia.” Such is the case of an electrical cable inserted in the urethra of a 12-year-old boy reported here, which is representative of this kind of impact.

## 1. Introduction

 Self-insertion of foreign bodies in the male urethra has been studied in detail in adults and less in children [1, 2]. Relevant issues taken under consideration on this subject are motivation, foreign object form, clinical presentation, complications, and management procedures [1–5]. The internet has captivated our daily life worldwide during the last two decades. Its influence led among others to changes in motivation and age presentation of foreign bodies' insertion in the urogenital tract. Childhood proves to be vulnerable to this effect, as this case report demonstrates.

## 2. Case Report

**Figure 1:**
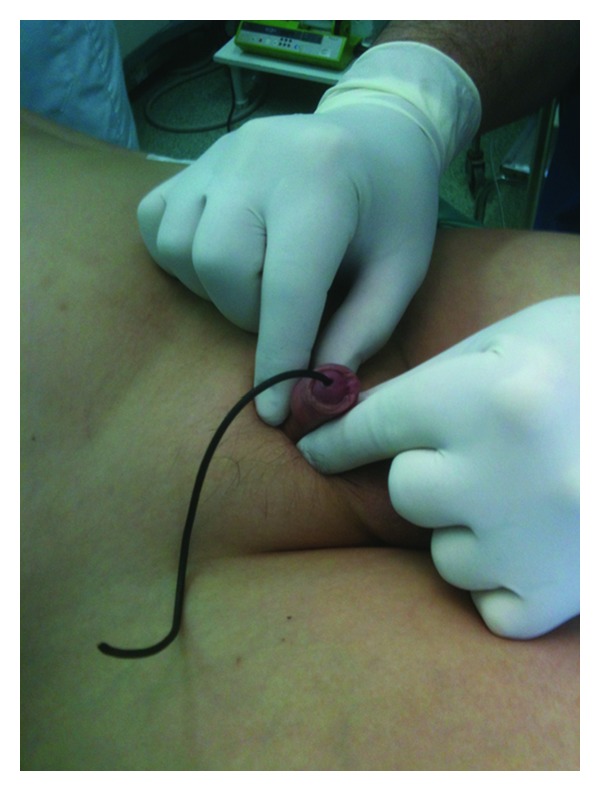
Clinical presentation of the inserted cable in the urethral meatus.

**Figure 2:**
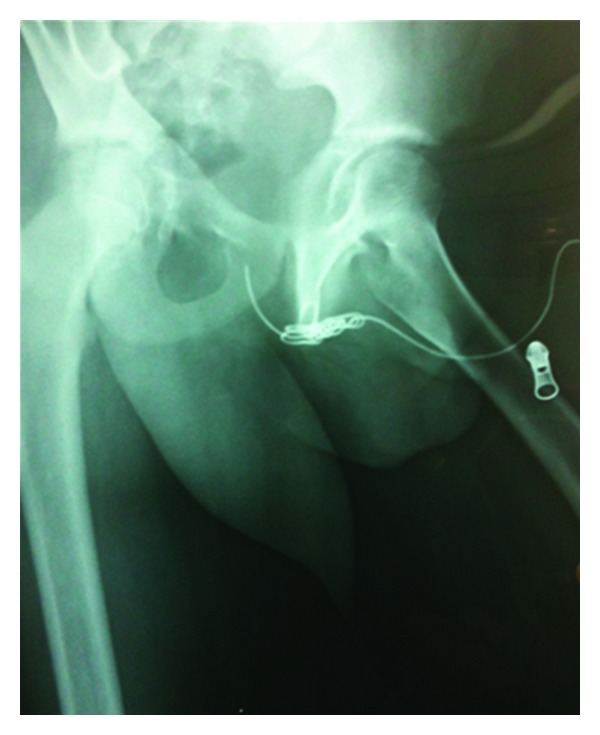
Pelvic plain radiography: twisted cable forming a coiled refractory structure in the posterior urethra.

 A 12-year-old male patient was admitted to the emergency department for urinary retention caused by self-insertion of an electrical television cable in his urethral meatus. The foreign body remained in the urethra for 48 hours before seeking medical help. During this period he was manipulating the cable, resulting in further insertion of the foreign body. When urine flow was blocked completely, he presented with pelvic and penile pain, bladder distention, and inability to void. The distal segment of the cable was protruding through the meatus (Figure 1). Pelvic plain radiography showed the long radiopaque metallic wire of the cable, which was twisted multiple times forming a coiled refractory structure (Figure 2). 

**Figure 3:**
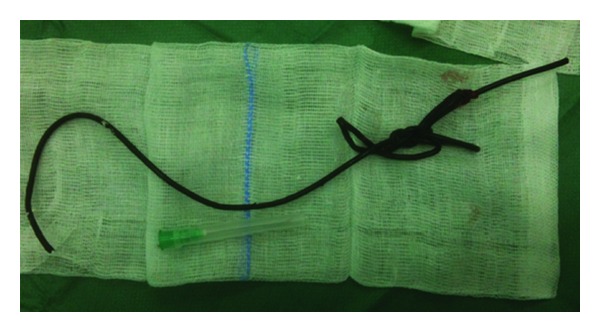
The foreign body (television cable) after extraction: Small amounts of tissue, characterized as inflammatory reaction were found at the refractory point.

 The symptoms were relieved after placement of a suprapubic bladder catheter. A quantity of 1200 mL urine was drained from the bladder. Cefuroxime and hyoscine butylbromide were administered intravenously. Percutaneous cystography through the catheter showed that the proximal end of the twisted cable was located into the posterior urethra without entering into the bladder. Removal of the foreign body through the urethral meatus was obtained by patient and gentle traction through the meatus under general anesthesia. A minor trauma of the urethra may have taken place as a slight amount of inflammatory tissue was found on the extracted catheter (Figure 3). A urethral Foley silicone catheter was placed in the urethra for 10 days. A normal urethrocystography was performed through the suprapubic catheter after removal of the urethral catheter. The suprapubic catheter was removed two days later. The patient during a six months follow-up period had a normal urine flow without late urethral stenosis. 

 During psychiatric consultancy he revealed that he had read in a website that if he inserted an electrical wire in his urethra and connected it with a battery to create electrical stimuli, he would achieve augmentation of penile length and experience simultaneously erotic satisfaction. There was not any mental of family disorder. As a son of an electrician, he had easy access to electrical stuff.

## 3. Discussion

 Self-insertion of foreign bodies in the male urogenital tract is an uncommon paraphilia studied in detail because of the variety it presents in many aspects [1–5]. There is a great diversity of the kind of objects inserted: sharp objects with refractory or tearing effect to the urethra (pins, pens, pencils, nails, bolts, toothbrushes, batteries, fishhooks, glasses, paper clips), objects that may coil around themselves (wire, electrical cables, chains, rubber bands), and biologic materials (vegetables, food cells, plant segments, bones) [1–5]. Electrical wire and cable insertion has been reported in adults [6, 7].

 The variety of objects, the way they are inserted, and the period they remain in the genitourinary tract affect clinical presentation [1–5]. Poor urinary stream, dysuria, swelling, urethral discharge, and urinary tract infection are the most common clinical signs [1, 3, 6, 7]. Hematuria, abscess formation, calculi, stricture, diverticulum, or erectile dysfunction are complications on late presentation [1, 2, 6]. An urethroperineal fistula is reported after 18-month stay of a golden chain in the urethra [3]. Direct removal, endoscopy, open operation of the bladder, and urethrotomy are methods of foreign body removal described [3, 4, 6–8]. Combinations of these methods have been described too [8].

 Reasons that cause self-insertion of objects are autoerotic stimulation, psychiatric disorders (dementia), self-mutilation, intoxication (cocaine), and curiosity [1–8]. Sometimes there is a combination of these causes. Behavior after insertion is particular; there is shame and embarrassment which often delay medical help, sometimes for years [3]. Identification of the true motivation is very important because recurrent insertions may occur, or in some cases there is a nonrecognized mental or organic disease which may involve even forensic implications [5, 9].

 The majority of the reported cases regard adulthood [1–5]. Kenney stated that genitourinary foreign body insertion by males has received “considerable attention in the worldwide urologic literature, little in the psychiatric literature, and none in the pediatric literature” [10]. Pediatric cases reported concerned normal children motivated mainly by curiosity or with mental defects [10]. However during the last ten years the internet created a new behavioral attitude. Children encounter certain issues that did not exist in television, books, and magazines previously; there is facility to immediate, inexpensive, timeless and private access to information and most importantly, with direct interaction with a specific website, or with other users. These characteristics upgrade the internet to a new pathogenic factor in inclining behavior of childhood and adolescence. All these characteristics were prominent in the case presented here; the boy was driven to electrical cable insertion after receiving information from a certain internet portal in an age of sexual immaturity. Sexuality is a field of major influence to this kind of behavior, and foreign body self-insertion into the genitourinary tract should not be regarded only a simple childish curiosity furthermore, but as a new causative category named “internet induced paraphilia.”
